# Semaphorin3A Signaling Is Dispensable for Motor Axon Reinnervation of the Adult Neuromuscular Junction

**DOI:** 10.1523/ENEURO.0155-17.2018

**Published:** 2018-05-16

**Authors:** Jennifer L. Shadrach, Brian A. Pierchala

**Affiliations:** 1 Department of Biologic and Materials Sciences, University of Michigan School of Dentistry, Ann Arbor, Michigan 48109; 2 Program in Cellular and Molecular Biology, University of Michigan School of Medicine, Ann Arbor, Michigan 48109

**Keywords:** Muscle Denervation, Neuromuscular Junction, Neuropilin1, Semaphorin3A

## Abstract

The neuromuscular junction (NMJ) is a specialized synapse that is formed by motor axon innervation of skeletal muscle fibers. The maintenance of motor-muscle connectivity is critical for the preservation of muscle tone and generation of movement. Injury can induce a robust regenerative response in motor axons, but severe trauma or chronic denervation resulting from neurodegenerative disease typically leads to inefficient repair and poor functional recovery. The axon guidance molecule Semaphorin3A (Sema3A) has been implicated as a negative regulator of motor innervation. Upon binding to a plexinA-neuropilin1 (Npn1) receptor complex, Sema3A initiates a downstream signaling cascade that results in axonal repulsion. Here, we established a reproducible nerve crush model to quantify motor nerve regeneration. We then used that model to investigate the role of Sema3A signaling at the adult NMJ. In contrast to previous findings, we found that *Sema3A* and *Npn1* mRNA decrease in response to denervation, suggesting that Sema3A-Npn1 signaling may regulate NMJ reinnervation. To directly test that hypothesis, we used inducible knockout models to ubiquitously delete Sema3A or Npn1 from adult mice. Despite demonstrating that we could achieve highly efficient gene deletion, disruption of Sema3A-Npn1 signaling did not affect the normal maintenance of the NMJ or disrupt motor axon reinnervation after a denervating injury.

## Significance Statement

Increased axonal growth and sprouting after a peripheral nerve injury are thought to signify the activation of a robust regenerative response that results in more efficient recovery. The axon guidance factor Sema3A has been proposed to generate a growth-inhibitory environment that reduces the regenerative capability of motor nerves. We sought to directly test how the inducible deletion of Sema3A or its receptor Npn1 alters muscle reinnervation after injury. Surprisingly, our work demonstrates that disruption of Npn1-Sema3A signaling does not alter reinnervation of the NMJ. Thus, the utility of targeting this pathway to improve recovery from denervating injuries may be more limited than suggested by earlier studies.

## Introduction

Complex motor function is achieved by communication between motor neurons and the skeletal muscle at a specialized synapse called the neuromuscular junction (NMJ). Maintenance of the NMJ requires the coordinated effort of three main structures: the presynaptic motor neuron terminal, the postsynaptic muscle apparatus, and terminal Schwann cells (TSCs). In healthy animals, a denervating injury produces a regenerative response in which both intrinsic and extrinsic factors act on motor nerve terminals to stimulate growth and axonal sprouting that result in subsequent muscle fiber reinnervation (Fawcett and Keynes, 1990; Chen et al., 2007). Because of their role in establishing target innervation during development, axon guidance molecules have been broadly proposed to regulate peripheral nerve regeneration after injury (Yaron and Zheng, 2007) and in response to neurodegenerative disease (Schmidt et al., 2009; Moloney et al., 2014).

One such axon guidance molecule that has been implicated in peripheral nerve regeneration is Semaphorin3A (Sema3A), which belongs to the large family of semaphorins that are defined by the presence of a conserved Sema domain at their amino-terminus (Yazdani and Terman, 2006). Class III semaphorins, including Sema3A, are secreted glycoproteins that signal through a multimeric receptor complex. For Sema3A, this receptor complex is composed of a class A Plexin (PlxA1-A4) and Neuropilin1 (Npn1). While PlxA receptors contain a large intracellular domain that initiates downstream signaling, the Npn1 receptor serves as a high-affinity binding partner that acts to stabilize the Plexin-Sema3 interaction (Janssen et al., 2012). Importantly, both PlxAs and Npn1 are absolutely required for Sema3A-mediated signal transduction (Takahashi et al., 1999; Rohm et al., 2000; Yaron et al., 2005).

Originally discovered as a chemorepellent that promotes sensory axon growth cone collapse (Luo et al., 1993; Messersmith et al., 1995), Sema3A has since been found to play a role in motor and sensory axon fasciculation and pathfinding during development (Behar et al., 1996; Taniguchi et al., 1997; Huber et al., 2005; Haupt et al., 2010), pruning of hippocampal axons (Bagri et al., 2003), inhibition of cortical axon collateral branching (Dent et al., 2004), and control of dendritic development (Morita et al., 2006; Shelly et al., 2011; Cheadle and Biederer, 2014). In the context of regeneration, Sema3A was found to be elevated after nerve crush injury (Scarlato et al., 2003; Ara et al., 2004; De Winter et al., 2006). Furthermore, upregulated *Sema3A* transcript specifically localized to fast type IIb/x muscle fibers, while slow type I/IIa muscle fibers did not express *Sema3A* (De Winter et al., 2006). Intriguingly, while fast and slow motor units have different metabolic and contractile properties (Kanning et al., 2010), they also exhibit well-documented differences in response to injury and neuropathology. Specifically, fast motor units are more susceptible to neurodegenerative diseases such as amyotrophic lateral sclerosis (ALS), spinal muscular atrophy (SMA), and even normal organismal aging (Frey et al., 2000). Additionally, fast muscle fibers exhibit less axonal sprouting and tend to repair poorly after injury, while slow fibers exhibit heightened axonal sprouting and repair more efficiently (Duchen, 1970; Lowrie et al., 1982; De Winter et al., 2006). Taken together, this previous work suggests a model in which the presence of Sema3A at the NMJ on fast muscle fibers generates a growth-inhibitory environment that may serve to reduce axonal sprouting and repair in response to injury.

In this study, we sought to directly test if there is a functional role for Sema3A signaling during reinnervation of the adult NMJ. To this end, we developed and characterized a highly reproducible nerve crush model that allows for the quantification of distinct phases of NMJ reinnervation. Then, given the essential role of Sema3A signaling during development, we generated inducible knockout mice to genetically delete Sema3A or Npn1 from all tissues of interest in adult mice. Contrary to the proposed role of Sema3A predicted by previous studies, we found that Sema3A signaling appears to be largely dispensable for normal NMJ reinnervation after injury.

## Materials and Methods

### Animals

All housing and procedures performed on mice were approved by the Institutional Animal Care and Use Committee of the University of Michigan. Wild-type C57BL/6J (000664), *Thy1^CreERT2-EYFP^* (012708), and *UBC^CreERT2^* (007001) mice were obtained from Jackson Laboratory. *Npn1^fx/fx^* (Gu et al., 2003) or *Sema3A^fx/fx^* (Riken Bioresource Center, RBRC01106) conditional mice were crossed with *UBC^CreERT2^* mice to generate *UBC^CreERT2^*;*Npn1^fx/fx^* (Npn1^UBC^) and *UBC^CreERT2^*;*Sema3A^fx/fx^* mice (Sema3A^UBC^). All mice were genotyped according to publicly available protocols except for Sema3A conditional mice. New primers (forward 5′-CACTGGGATTGCCTGTCTTT-3′ and reverse 5′-ACGGAGCAAGCACACAGCTA-3′) were designed to detect a 363-bp wild-type band and/or a 400-bp mutant band. For all experiments, both male and female mice were analyzed in similar numbers.

### Conditional deletion

**Figure 1.**
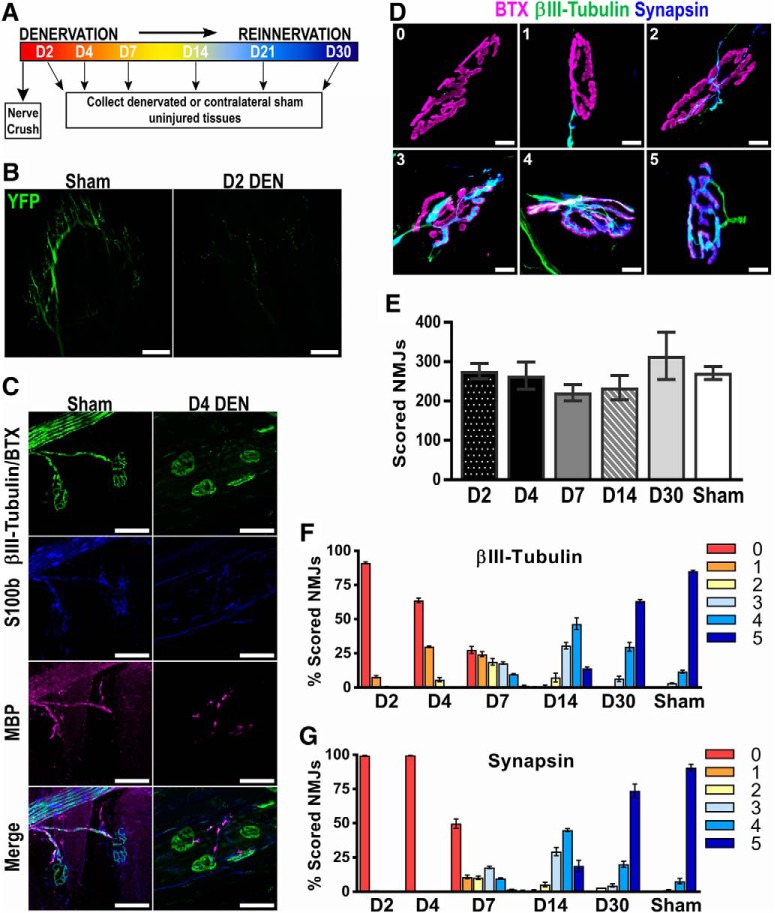
Characterization of the common peroneal crush methodology. ***A***, Graphical depiction of the experimental paradigm. A common peroneal nerve crush and contralateral sham operation were performed at day 0 (D0). Denervated (Den) and sham EDLs were collected at various time points following injury (*n* = 3 for all time points except D30, *n* = 2). ***B***, Representative whole-mount immunofluorescent images of sham (left) and D2 denervated (right) EDL sections from *Thy1*-YFP mice demonstrate a total loss of nerve innervation following the common peroneal nerve crush. Scale bar represents 250 µm. ***C***, Myelination at the D4 time point following nerve crush was examined by immunofluorescent staining. βIII-Tubulin and acetylcholine receptors stained with bungarotoxin (BTX) are shown in green, while the myelin markers S100 calcium binding protein B (S100b) and myelin basic protein (MBP) are shown in blue and magenta, respectively. Scale bar represents 50 µm. ***D***, Representative images illustrate the scoring system (0–5) used to quantify NMJ reinnervation. BTX marks the endplate (magenta), βIII-tubulin allows for visualization of the motor nerve (green), and synapsin stains the presynaptic nerve terminal (blue). Scale bar represents 10 µm. ***E***, The total number of neuromuscular junctions (NMJs) analyzed per animal was consistent across all time points analyzed (n.s., one-way ANOVA). ***F***, ***G***, Reinnervation was analyzed over the 30-d time course, and the percentage of NMJs scored as 0–5 is shown. Scoring was performed based on both nerve βIII-tubulin staining (***F***) and presynaptic synapsin staining (***G***). Error bars represent the mean ± SE.

Tamoxifen (TMX, Sigma-Aldrich) was administered at a dose of 0.25 mg/g body weight to Npn1^UBC^ or Sema3A^UBC^ mice by oral gavage once a day for 5 d. Mice were then given a resting period of 12–15 d to allow for complete Cre-mediated recombination before performing nerve crush experiments (Fig. 1). In all experiments, two types of control littermates were used: (1) *Cre* wild-type (*UBC^CreERT2^* negative mice treated with TMX) and (2) vehicle only [*UBC^CreERT2^* mice treated with corn oil (CO)]. For the Npn1-conditional deletion, both control groups behaved similarly and therefore all results were averaged and displayed as one wild-type control group (Npn1^WT^). For the Sema3A deletion, CO-treated mice exhibited a partial reduction in *Sema3A* transcript levels (Fig. 5*A*), so only *Cre* wild-type mice (Sema3A^WT^) were used.

### Common peroneal nerve crush

Mice were deeply anesthetized with 2%–3% isoflurane mixed with oxygen, and the surgical site was shaved and disinfected. A small incision was made along the lateral aspect of the distal hindlimb from just above the ankle to just below the knee. A dissecting microscope was used to expose the common peroneal nerve through a small opening between the anterior compartment (tibialis anterior) and the posterior compartment (lateral gastrocnemius). The exposed nerve was then crushed by applying pressure with a pair of forceps for 40 s. Following the crush procedure, the site where the anterior and posterior muscles were separated was closed with a suture, and then the skin was sutured at the incision site. In a small number of experiments, a nerve cut was performed instead of a nerve crush. In these instances, the same procedure was performed except that a 3–5-mm piece of nerve was excised as opposed to being crushed with forceps.

### RNA isolation and RT-qPCR

Tissues were harvested and snap-frozen in liquid nitrogen. Homogenization was performed in TRIzol reagent (Thermo Fisher Scientific) using a TissueLyser II (Qiagen) set at 30 Hz for 5 min. Samples were then centrifuged at 12,000 × *g* for 10 min at 4°C. RNA was isolated by phase separation with chloroform followed by isopropanol precipitation (per the manufacturer’s protocol). In some instances, RNA was isolated with the Direct-zol RNA MiniPrep Kit (Zymo Research) according to manufacturer guidelines. The concentration of isolated RNA was determined by NanoDrop (Thermo Fisher Scientific), and 1 µg cDNA was synthesized using the Superscript III First-Strand Synthesis SuperMix kit (Invitrogen). RT-qPCR was performed using the 7900HT Fast Real-Time PCR System (Applied Biosystems) with the appropriate primer set (Table 1) and FastStart Universal SYBR Green Master Mix (Roche). Primer sequences were obtained through the Harvard Primer bank, Primer3, or from previously published literature (Tan et al., 2011; Fukuda et al., 2013). B2M was used as an internal control for all dCT calculations, and gene expression was calculated relative to the control condition.

**Table 1.** Primer sequences used for real-time RT-PCR

**Gene**	**Forward Primer (5′–3′)**	**Reverse Primer (5′–3′)**	**BP**	**Source**
B2M	TTCTGGTGCTTGTCTCACTGA	CAGTATGTTCGGCTTCCCATTC	104	Tan et al., 2011
Npn1	GAGGACAGAGACTGCAAGTATG	CTGAAGACACCACAGGAGAAG	115	Primer3
Sema3A	ATATGCAAGAATGACTTTGGTGGAC	AAGGAACACCCTTCTTACATCACTC	258	Primer3
GAP43	ATAACTCCCCGTCCTCCAAGG	GTTTGGCTTCGTCTACAGCGT	201	Harvard PrimerBank
MuSK	CCTCAGCCCGAGATTTCTTGG	GTCTTCCACGCTCAGAATGGT	111	Harvard PrimerBank
P0	CATCTCTTTTACCTGGCGCTAC	TGTAAGGTTGTCCCTTGGCATA	83	Harvard PrimerBank
S100	CTTCCTGGAGGAAATCAAGGAG	CTCATGTTCAAAGAACTCATGGC	148	Primer3
PlexinA1	GAGTGCAAGGAAGCTTTTGC	TCCTCAATCCCAGGAAACAG	131	Fukuda et al., 2013
PlexinA2	TATAACTGCAGTGCCCACCA	TGGGGACAGTCCTCTGAAAC	149	Primer3
PlexinA3	AGCATTCTGTGGTTTTCATCG	CACCTGCTTCTCACTCAGGA	179	Primer3
PlexinA4	ATCTAGAGTGGCGACAAGGAAG	TGGAGACAGTGGAGTTGTTCAC	189	Fukuda et al., 2013
MYH1	CTCTTCCCGCTTTGGTAAGTT	CAGGAGCATTTCGATTAGATCCG	187	Harvard Primer Bank
MYH2	ACTTTGGCACTACGGGGAAAC	CAGCAGCATTTCGATCAGCTC	155	Harvard Primer Bank
MYH3	CCAAAACCTACTGCTTTGTGGT	GGGTGGGTTCATGGCATACA	149	Harvard Primer Bank
MYH4	CTTTGCTTACGTCAGTCAAGGT	AGCGCCTGTGAGCTTGTAAA	139	Harvard Primer Bank
MYH6	TGCACTACGGAAACATGAAGTT	CGATGGAATAGTACACTTGCTGT	204	Harvard Primer Bank
MYH7	GCTACGCTTCCTGGATGATCT	CCTCTTAGTGTTGACAGTCTTCC	248	Harvard Primer Bank

### Npn1 immunoprecipitation

Muscle or spinal cord samples were dissected and placed into immunoprecipitation buffer [10% glycerol, C0mplete protease inhibitors (Roche), and sodium vanadate in Tris-buffered saline (TBS), pH 6.8]. Tissues were homogenized with the Tissue Lyser II (Qiagen) set at 30 Hz for 5 min. Samples were then detergent extracted by the addition of nonidet *p*-40 (1% final concentration) followed by constant rotation for 30 min at 4°C, and insoluble material was removed by centrifugation for 5 min at maximum speed in a Microfuge. Before Npn1 immunoprecipitation (IP), samples were first precleared with a mixture of protein A/G agarose beads (Roche) for 1 h. Next, a control IP was performed in which samples were incubated with a nonspecific goat control antibody and protein A/G beads for 2 h. Finally, the Npn1 IP was performed overnight with a Npn1 antibody (AF566, R&D Systems) and protein A/G beads. For all incubations, samples were left under constant rotation at 4°C. The next day, samples were lightly centrifuged (5000 rpm, 5 min), and the supernatants were collected and denatured in 2× sodium dodecyl sulfate (SDS) sample buffer (20% glycerol, 4% SDS, 1% β-mercaptoethanol, and bromophenol blue in TBS, pH 6.8) by heating for 10 min at 100°C. Meanwhile, the immunoprecipitated products were washed three times with immunoprecipitation buffer followed by denaturation in the same manner as described for the supernatants.

### Protein isolation for Sema3A immunoblotting

We used many different methods of protein isolation and various Sema3A antibodies to try to demonstrate effective knockdown of Sema3A protein in Sema3A^UBC^ mice. However, most methods resulted in obtaining a nonspecific protein band in the reported 95–105-kDa molecular weight range. The only methodology that we found to be successful was when protein was copurified with RNA using the Direct-zol RNA MiniPrep Kit (Zymo Research). With this kit, after RNA binds to the RNA-binding cup, protein in the flow-through was precipitated with ice-cold acetone for 30 min on ice. Samples were then centrifuged for 10 min at max speed to pellet the protein precipitate. The protein was then washed with 100% ice-cold ethanol, centrifuged again, and resuspended in water. Finally, the protein was denatured by adding an equal volume of 2× SDS sample buffer and heating for 10 min at 100°C.

### Immunoblotting

All samples were resolved on a 7% SDS-PAGE gel and transferred to polyvinylidene fluoride (PVDF) membranes. Membranes were blocked in 4% milk in TBS-T (TBS, pH 7.4, and 0.1% Tween 20) for 1 h at room temperature. Primary antibodies [α-tubulin (1:30,000, T9026, Sigma-Aldrich), actin (1:1000, SC-1616-G, Santa Cruz Biotechnologies), Npn1 (1:1000, AF566, R&D Systems), or Sema3A (1:1000, ab23393, Abcam)] were diluted in 3% bovine serum albumin (BSA) and incubated overnight at 4°C. The following day, membranes were washed and incubated with appropriate horseradish peroxidase (HRP)-linked secondary antibodies (1:10,000) in 3% BSA (Jackson ImmunoResearch) followed by visualization with a chemiluminescent substrate (Thermo Fisher Scientific).

### Tissue preparation

EDL muscles were fixed in 4% paraformaldehyde (Electron Microscopy Sciences) for 10 min at room temperature. Tissues were then washed 3× 20 min in PBS and soaked overnight in 30% sucrose at 4°C. Muscles were embedded in O.C.T compound (Tissue-Tek) and frozen at –80°C. 50-µm longitudinal cryosections were cut using a CM1950 cryostat (Leica Biosystems) such that 3–5 sections ∼300 µm apart were placed on one slide.

### Immunostaining

Sections were rehydrated in PBS and then permeabilized/blocked in 0.3% Triton X-100, 1% BSA, 10% donkey serum (Jackson ImmunoResearch), and MOM blocking reagent (Vector Laboratories) for 1 h at room temperature. Slides were then incubated in primary antibodies [anti-synapsin-1 (5297S, Cell Signaling), anti-β-tubulin III (T8578, Sigma-Aldrich), anti-S100 (RB044A0, Thermo Scientific Lab Vision), anti-myelin basic protein (AB9348, EMD Millipore)] in 0.3% Triton X-100, 1% BSA overnight at 4°C. The following day, sections were washed in PBS and stained with fluorescently conjugated α-bungarotoxin and/or appropriate secondary antibodies (Biotium) in 0.3% Triton X-100 for 1 h at room temperature. After final PBS washes, slides were coverslipped with DAPI mounting medium (Southern Biotech) and imaged on a confocal microscope (Leica SP5).

### Reinnervation analysis

Endplates identified by bungarotoxin staining were imaged at 20× magnification with high resolution (2048 × 2048) and a *z*-step size of 1.5 µm. Every endplate on one slide containing 3–5 sections was imaged. Maximum projection (LAS Software, Leica Biosystems) was applied to all files, and every in-plane NMJ was scored from 0 to 5 to reflect its innervation status. Two markers were used for innervation scores: (1) β-tubulin III to reflect nerve reinnervation and (2) synapsin to reflect presynaptic differentiation. All imaging and scoring was performed by a single blinded observer that was unaware of the strain, genotype, and time point being analyzed.

### Muscle force measurement

Mice were anesthetized with an initial intraperitoneal injection of avertin (tribromoethanol, 250 mg/kg), and supplemental injections were given as needed to maintain an adequate level of anesthesia during the procedure. Isometric contractile properties were measured *in situ* similarly to Gumerson et al. (2013). The common peroneal nerve and distal third of the EDL muscle were carefully exposed without damaging muscle, nerve, or blood vessels during the dissection. The mouse was placed on a platform maintained at 37°C, and the hindlimb was immobilized by pinning through the knee and taping the foot to the platform. The distal tendon of the EDL was severed and then tied to the lever arm of a servomotor (300C-LR-FP, Aurora Scientific) using 5-0 braided silk suture. A continual drip of saline warmed to 37°C was administered to the muscle to maintain temperature. Platinum electrodes placed on either side of the midbelly of EDL provided direct muscle stimulation, while a pair of platinum wire electrodes placed under the peroneal nerve provided nerve stimulation. The muscle was adjusted to the optimum length, and maximum twitch force was found for both direct muscle and nerve stimulation. Maximum isometric tetanic force was then determined by delivering a train of constant-current pulses at a frequency of 220 Hz for 300 ms, first directly to the muscle and then subsequently to the nerve. Each current pulse was 0.2 ms in duration and was exceeded by ∼25% of the intensity required to elicit a maximum twitch response from each tissue, respectively. Custom-designed software (LabVIEW 2014; National Instruments) controlled stimulus pulses and recorded force responses. The sequence of direct muscle stimulation followed by nerve stimulation was repeated an additional 3 times for a total of 4 maximum-force measurements. Maximum isometric tetanic force (P_o_) for both muscle and nerve stimulation was then defined as the average of the 4 trials. Optimal muscle length (L_o_) was measured with digital calipers, and the weight of the muscle was recorded. Muscle fiber length (L_f_) was determined by multiplying the EDL muscle length by 0.44, a previously established L_f_-to-L_o_ ratio (Brooks and Faulkner, 1988). The physiologic cross-sectional area (PCSA) was then estimated by dividing muscle mass by the product of L_f_ and the density of mammalian skeletal muscle (1.06 g/cm^3^). Specific force was then calculated by normalizing P_o_ to the PCSA.

### Statistical analysis

All statistical analyses were performed using Prism 7 software (GraphPad). One- or two-way ANOVA was used for all analyses. Significant differences among pairwise comparisons were identified by Tukey’s or Sidek’s *post hoc* tests (Table 2). All graphs and error bars represent the mean ± standard error (SE).

**Table 2.** Statistical analysis

**Figure**	**Data structure**	**Type of test**	***Post hoc* test**
1*E*	Normal distribution	One-way ANOVA	Tukey
2*A*	Normal distribution	Repeated-measures two-way ANOVA	Tukey
2*B*	Normal distribution	Repeated-measures one-way ANOVA	Tukey
2*C*	Normal distribution	Repeated-measures two-way ANOVA	Tukey
2*D*	Normal distribution	Repeated-measures two-way ANOVA	Tukey
3*A–G*	Normal distribution	One-way ANOVA	Tukey
4*B*	Normal distribution	Two-way ANOVA	Sidek
4*E*	Normal distribution	Two-way ANOVA	Tukey
4*F*,*G*	Normal distribution	Two-way ANOVA	Tukey
4*L*	Normal distribution	Two-way ANOVA	Tukey
4*M*	Normal distribution	Two-way ANOVA	Tukey
5*A*	Normal distribution	Two-way ANOVA	Sidek
5*C*	Normal distribution	Two-way ANOVA	Tukey
5*H*	Normal distribution	Two-way ANOVA	Tukey
5*I*	Normal distribution	Two-way ANOVA	Tukey

## Results

### Characterization of the common peroneal crush methodology

To directly examine whether Sema3A signaling plays a role in NMJ reinnervation, we first established a reproducible nerve crush model that allowed for the quantification of distinct phases of NMJ reinnervation. To this end, we performed a nerve crush on the common peroneal branch of the sciatic nerve that innervates the anterior muscles of the distal hindlimb. This approach allowed us to examine reinnervation in the EDL, which is a thin muscle amenable to systematic and thorough analysis. Additionally, from previously published studies, we could also predict the approximate time course of initial muscle denervation and subsequent reinnervation (Magill et al., 2007; Bauder and Ferguson, 2012; Dalkin et al., 2016). The experimental design we used to fully characterize this nerve crush model is illustrated in Fig. 1*A*. Briefly, a common peroneal nerve crush and a contralateral sham surgery were performed on wild-type mice. Injured and control EDL muscles were then collected at different time points (days 2–30) and processed to systematically collect longitudinal sections from the entire muscle (see Materials and Methods). Immunostained sections were then analyzed to quantify the extent of NMJ denervation or reinnervation.

To ensure that the nerve crush injury induced a complete degeneration of motor axons from the EDL muscle, we used *Thy1^CreERT2-EYFP^* mice that exhibit strong YFP expression in peripheral nerves. Sham-injured and denervated EDLs were collected 2 d after nerve crush, and whole-mount imaging was used to broadly examine denervation after injury. As expected, intact YFP^+^ innervation was observed in sham-injured EDLs, while a dramatic and uniform loss of YFP signal was apparent 2 d after nerve crush (Fig. 1*B*). In addition to loss of innervation, we also examined myelination of motor axons after common peroneal nerve crush (Fig. 1*C*). Day 4 (D4) sham-injured and denervated EDLs were stained with a combination of bungarotoxin (BTX) and βIII-tubulin (green), S100 calcium binding protein (S100b, blue), and myelin basic protein (MBP, magenta). In sham-injured muscle, BTX labeled endplates were innervated by βIII-tubulin^+^ motor nerves that exhibited normal myelination as indicated by S100b and MBP staining (Fig. 1*C*, left panels). In contrast, at D4 after nerve crush, withdrawal of βIII-tubulin^+^ motor nerves was accompanied by a marked reduction of S100b, and only fractured pockets of MBP staining was evident (Fig. 1*C*, right panels).

After demonstrating that this injury model produced a reproducible and uniform denervation, we devised a scoring system to quantify NMJ reinnervation. BTX (magenta) was used to identify the endplate region, while βIII-tubulin (green) and synapsin (blue) were used as markers for nerve reinnervation and presynaptic differentiation, respectively. Six different morphologic categories were created to reflect the innervation status of individual NMJs (Fig. 1*D*): completely denervated (score 0); motor nerve is approaching, but not innervating, an endplate (score 1); <50% of the endplate area is innervated (score 2); >50% of the endplate area is innervated (score 3); motor axon extends throughout the endplate, but does not fill the entire space (score 4); and completely reinnervated (score 5).

Next, we applied this scoring system to quantify reinnervation in C57BL/6 wild-type mice at various time points following nerve crush. Importantly, the total number of individual NMJs analyzed was consistent across all time points, with ∼200–300 in-plane NMJs analyzed per mouse (Fig. 1*E*). Furthermore, using βIII-tubulin (Fig. 1*F*) and synapsin (Fig. 1*G*) staining to quantify the percentage of NMJs scored as 0–5 demonstrated that this scoring system could identify unique phases of denervation and reinnervation throughout the time course. At D2 after injury, almost all NMJs were completely denervated and received a score of 0. By D4, early motor nerve reinnervation at some NMJs was evident, but no presynaptic differentiation was observed (all synapsin-based scores were 0). From D7 to D14, increasingly more NMJs became reinnervated, while from D14 to D30, maturation of the innervating motor nerve was observed as scores shifted from 3 or 4 to an increasing number of 5s. Although innervation at D30 began to approach that observed in sham-injured EDLs, full recovery was not yet histologically apparent. Taken together, our data demonstrate that this common peroneal nerve crush model is sufficient to carefully interrogate motor axon regeneration, synaptogenesis, and remyelination after nerve injury.

### Sema3A signaling family gene expression

**Figure 2.**
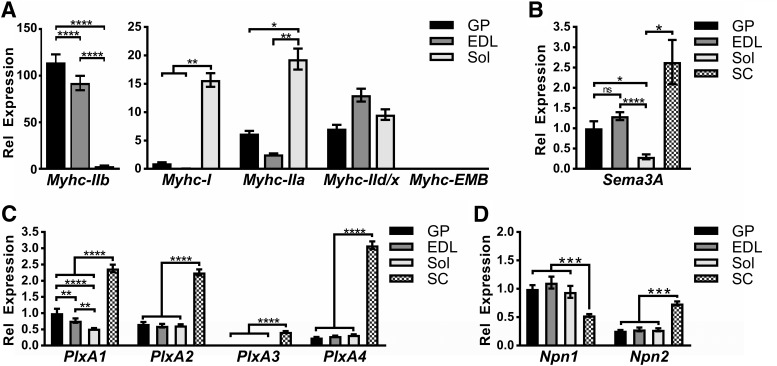
Sema3A and its receptor components are expressed in skeletal muscle and spinal cord. Quantitative real-time PCR (RT-qPCR) was performed on RNA isolated from the gastrocnemius/plantaris (GP), extensor digitorum longus (EDL), soleus (Sol), and spinal cord (SC) tissues of C57BL/6J mice (*n* = 6). Individual gene expression was normalized to the *β2-Microglobulin* (*B2M*) housekeeping gene. Myosin heavy chain (MyHC) isoforms (***A***), *Semaphorin3A* (*Sema3A*, ***B***), class A plexin family members (*PlxA1-4*, ***C***), and neuropilin family members (*Npn1-2*, ***D***) were examined. Error bars represent the mean ± SE. *, *p* ≤ 0.05; ** *p* ≤ 0.01; ***, *p* ≤ 0.001; **** *p* ≤ 0.0001.

Previous work examining Sema3A signaling at the NMJ found that *Sema3A* mRNA was not detectable in normally innervated muscle. However, an upregulation of *Sema3A* was observed after nerve crush injury in the fast-twitch fiber types of the gastrocnemius (GP) muscle, while the slow-twitch fibers of the soleus (Sol) muscle showed no response (De Winter et al., 2006). Similar to the GP, the EDL is predominantly composed of fast-twitch fibers. Therefore, we reasoned that patterns of Sema3A signaling should be similar in the two muscles. To directly examine this, we isolated RNA from wild-type GP, Sol, and EDL muscles and from the spinal cord (SC). RT-qPCR was then used to analyze *Sema3A* and its signaling partners (Fig. 2). First, as a control, we examined the expression of different isoforms of myosin heavy chain (MyHC) that are characteristic to fast- or slow-twitch fiber types (Agbulut et al., 2003). As expected, the Sol muscle exhibited a strong enrichment in the slow myosin isoforms *Myhc-I* (GP, *p* = 0.006; EDL, *p* = 0.0033) and *Myhc-IIa* (GP, *p* = 0.015; EDL, *p* = 0.0016), while the fast isoform *Myhc-IIb* was dramatically upregulated in the GP (*p* < 0.0001) and the EDL (*p* < 0.0001). Furthermore, a similar level of *Myhc-IId/x* was observed in all three muscle groups, and the embryonic Myhc (*Mych-EMB)* was barely detectable in any of the adult muscles (Fig. 2*A*).

Once we established that proper *Myhc* isoform profiles were expressed in the different muscle groups, we examined the expression levels of *Sema3A*, *PlxA*, and *Npn* transcripts. In contrast to what had been previously reported (De Winter et al., 2006), we could reproducibly detect *Sema3A* mRNA in uninjured adult skeletal muscle (Fig. 2*B*). More specifically, we found that *Sema3A* was expressed at a similar level in the GP (1.00 ± 0.17), EDL (1.30 ± 0.01), and SC (2.635 ± 0.55), while a significant reduction of transcript was observed in the slow-twitch Sol (0.295 ± 0.06) muscle compared to the GP (*p* = 0.014), EDL (*p* < 0.0001), and SC (*p* = 0.019). Additionally, analysis of *PlxA1-A4* (Fig. 2*C*) and *Npn1-2* (Fig. 2*D*) family members revealed that all receptor components were expressed at similar levels within the different muscles examined, except for *PlxA1*, which had the highest expression in the GP and lowest expression levels in the Sol.

**Figure 3.**
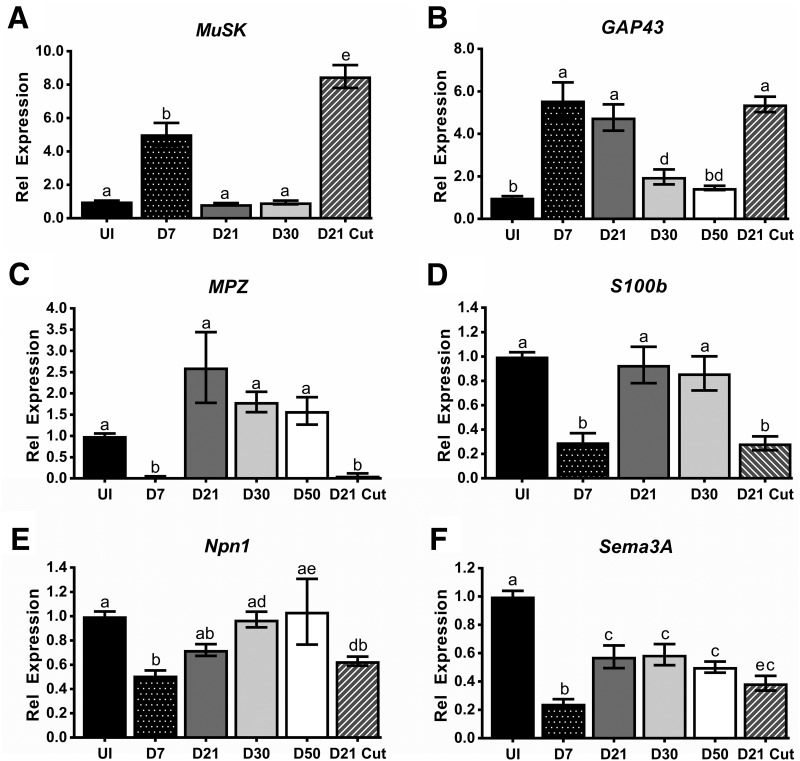
*Sema3A* and *Npn1* gene expression decrease in the EDL following common peroneal nerve crush. A common peroneal nerve crush was performed and RNA was isolated at D7 (*n* = 9), D21 (*n* = 9), D30 (*n* = 4), and D50 (*n* = 4) from denervated and the contralateral control EDL muscles. In some cases, a nerve cut was performed instead of a nerve crush, and tissues were collected at the D21 time point (*n* = 8). ***A–D***, Gene expression for the postsynaptic gene *Muscle-specific kinase* (*MuSK,* ***A***), the regeneration marker *Growth-associated protein 43* (*GAP43*, ***B***), and two myelin markers *Myelin protein zero* (*MPZ,* ***C***) and *S100 calcium binding protein B (S100B*, ***D****)* were used as controls to demonstrate the effectiveness of the nerve crush and how the regenerative response changes over the examined time course. ***E***, ***F***, Gene expression for *Neuropilin1* (*Npn1,* ***E***) and *Semaphorin3A* (*Sema3A,* ***F***) decrease in response to nerve crush injury. *Npn1* levels return to baseline over the time course, while Sema3A remains low even at the latest time point examined. For all graphs, error bars represent the mean ± SE, and letters represent significant differences (a–b, a–e: *p* < 0.0001; b–c: *p* ≤ 0.001; a–d, b–d: *p* < 0.01; b–e, a–c: *p* < 0.05).

To examine if *Npn1* and *Sema3A* transcript levels change in response to a denervating injury, a common peroneal nerve crush was performed on wild-type mice. Uninjured (UI) and denervated EDLs were then collected at different time points (D7–D50) after injury. In some cases, a nerve cut was performed instead of a nerve crush, and tissue was collected at D21 to examine how gene expression was altered in the absence of reinnervation. Several control genes were used to monitor the progression of the degenerative and regenerative response to the nerve crush. Muscle-specific kinase (MuSK), one of the main components of the postsynaptic apparatus, and growth-associated protein 43 (GAP43), a gene associated with regenerating axons and Schwann cells, are known to be induced by denervating injuries (Valenzuela et al., 1995; Bowen et al., 1998; Xu et al., 2008; Li et al., 2010). Conversely, myelin protein zero (MPZ) and S100b have been reported to be reduced following nerve crush (Gupta et al., 1988; Mitchell et al., 1990; Magill et al., 2007; Li et al., 2010). As in these previous studies, the common peroneal nerve crush model led to similar changes in gene expression. *MuSK* was significantly upregulated at D7 after nerve crush (UI, 1.00 ± 0.06; D7, 5.03 ± 0.69, *p* < 0.0001), but returned to baseline by D21 (D21, 0.84 ± 0.07, n.s.). Furthermore, preventing reinnervation resulted in a prolonged upregulation of *MuSK* mRNA (8.50 ± 0.69, *p* < 0.0001) at the D21 time point (Fig. 3*A*). Similarly, a robust induction of *GAP43* mRNA was observed at D7 (UI, 1.00 ± 0.07; D7, 5.57 ± 0.86, *p* < 0.0001) and when reinnervation was prevented (D21 cut, 5.39 ± 0.3654, *p* < 0.0001); however, it exhibited a slower return to baseline uninjured levels (D21, 4.77 ± 0.62; D30, 1.98 ± 0.35; D50, 1.46 ± 0.10) over the 50-d time course (Fig. 3*B*). Both *MPZ* (Fig. 3*C*) and *S100b* (Fig. 3*D*) exhibited a similar change in gene expression after denervation. In both cases, a significant reduction in gene expression was observed at the D7 time point (MPZ: UI, 1.00 ± 0.06, D7, 0.03 ± 0.02, *p* < 0.0001; S100b: UI, 1.00 ± 0.04, D7, 0.30 ± 0.08, *p* < 0.0001), with levels normalizing to that observed in the uninjured EDL by D21 (MPZ: D21, 2.61 ± 0.83; S100b: D21, 0.93 ± 0.15). Additionally, the restoration of *MPZ* and *S100b* mRNA levels at the D21 time point could be prevented by prolonged denervation after nerve cut (MPZ: 0.06 ± 0.06, *p* < 0.0001; S100b: 0.29 ± 0.6, < 0.0001).

Given that *Sema3A* transcript levels are similar among uninjured GP and EDL muscles, evidence from previous studies suggested that common peroneal nerve crush would induce enhanced *Sema3A* expression (Scarlato et al., 2003; Ara et al., 2004; De Winter et al., 2006). However, instead we observed a significant reduction in both *Npn1* (UI, 1.00 ± 0.04, D7, 0.51 ± 0.04, *p* < 0.0001) and *Sema3A* (UI, 1.00 ± 0.04, D7, 0.24 ± 0.03, *p* < 0.0001) at the D7 time point (Fig. 3*E*,*F*). By D30 after the initial injury, *Npn1* levels returned to baseline (0.97 ± 0.06), while *Sema3A* mRNA levels partially rebounded by D21 after injury (0.57 ± 0.08, *p* = 0.0001), but remained significantly reduced compared to the uninjured control muscle throughout the entire time course (D30, 0.59 ± 0.08, *p* = 0.04; D50, 0.50 ± 0.04, *p* = 0.003). Altogether, these results suggest that there is decreased Sema3A signaling in response to denervation. Despite these unexpected findings, whether Sema3A signaling plays a functional role in the process of reinnervation remained an unanswered question.

### Direct examination of Npn1-Sema3A signaling during motor reinnervation of the NMJ

**Figure 4.**
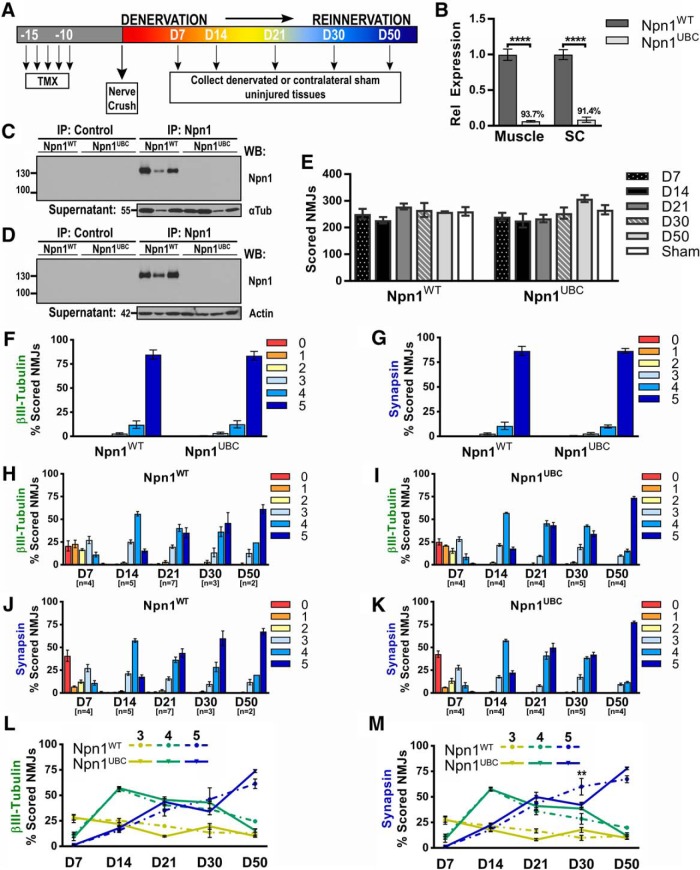
Loss of Npn1 modestly impairs NMJ reinnervation after injury. ***A***, Graphical depiction of the experimental paradigm. Tamoxifen (TMX) was administered for 5 d, followed by an at least 10-d rest period to allow for Cre-mediated recombination. At D0, a common peroneal crush was performed, and tissues were collected at the various time points. ***B***, RT-qPCR was used to assess the level of *Npn1* knockdown in GP muscle and SC from all mice used to examine reinnervation in subsequent experiments. The level of *Npn1* transcript detected in Npn1^UBC^ mice (*n* = 21) is graphed relative to that observed in Npn1^WT^ control littermates (*n* = 20), with the average percentage knockdown in Npn1^UBC^ mice displayed above the bar graph. ***C***, ***D***, Control or Npn1 IP, followed by immunoblotting for Npn1, was used to assess the amount of Npn1 protein in Npn1^WT^ and Npn1^UBC^ mice after TMX-mediated deletion. Representative Western blots from the D30 time point demonstrate that Npn1^UBC^ mice have no detectable levels of Npn1 protein in either the GP muscle (***C***) or the whole SC (***D***). Supernatants immunoblotted for α-tubulin (αTub) or actin are provided as loading controls. ***E,***Npn1 deletion does not alter the total number of scored NMJs observed at different time points after nerve crush injury (n.s., one-way ANOVA). ***F***, ***G***, The innervation status of sham-operated uninjured EDLs from Npn1^UBC^ (*n* = 4) and Npn1^WT^ (*n* = 4) mice was examined at the D21 time point. Scoring performed on the basis of nerve fiber (βIII-tubulin, ***F***) or presynaptic terminal (synapsin, ***G***) staining demonstrates that loss of Npn1 does not perturb the normal maintenance of the NMJ. ***H–K***, Reinnervation following a nerve crush injury was examined in Npn1^WT^ littermate controls (***H***, ***J***) and Npn1^UBC^ (***I***, ***K***) mice. Innervation scores based on nerve βIII-tubulin staining (***H***, ***I***) or presynaptic synapsin staining (***J***, ***K***) produced similar trends. Despite loss of Npn1, early reinnervation (D7–D21) occurs normally. However, at D30, there appears to be a delay in motor nerve maturation as the number of NMJs scored as a 5 are decreased. This effect is only transient, as reinnervation is largely complete by the D50 time point. ***L***, ***M***, Reinnervation data were replotted using only scores 3–5 to directly compare differences between the two genotypes. Error bars represent the mean ± SE. **** *p* ≤ 0.0001, **, *p* < 0.01.

**Figure 5.**
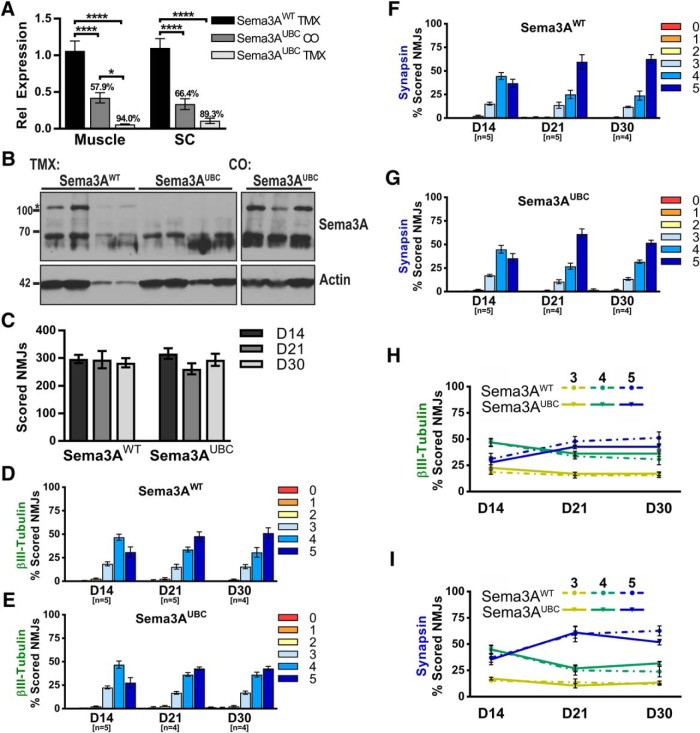
Loss of Sema3A does not impact NMJ reinnervation after injury. ***A***, RNA was isolated from the GP muscle and SC of mice used for subsequent reinnervation studies. The level of *Sema3A* transcript detected in Sema3A^UBC^ mice treated with corn oil (*n* = 10) or TMX (*n* = 12) is graphed relative to that observed in Sema3A^WT^ control littermates (*n* = 12). The average percentage knockdown in Sema3A^UBC^ mice is displayed above each bar graph. ***B***, GP muscle lysates were isolated from Sema3A^WT^ and Sema3A^UBC^ mice at various time points after nerve crush (D14–D30) and subjected to Western blotting. No detectable Sema3A protein was observed after TMX-mediated deletion in Sema3A^UBC^ mice. Supernatants immunoblotted for actin are provided as a loading control. ***C***, Loss of Sema3A does not alter the number of NMJs scored at different time points (n.s., one-way ANOVA). ***D–G***, Reinnervation after a common peroneal crush was analyzed in Sema3A^WT^ control littermates (***D***, ***F***) and Sema3A^UBC^ (***E***, ***G***) mice. Innervation scores based on nerve βIII-tubulin staining (***D***, ***E***) or presynaptic synapsin staining (***F***, ***G***) followed similar trends. No differences in reinnervation were detected at any time point. (***H***, ***I***) Reinnervation data were replotted using only scores 3–5 to directly compare differences between the two genotypes. Error bars represent the mean ± SE. **** *p* ≤ 0.0001, *, *p* < 0.05.

Previous studies have localized *Sema3a* mRNA expression to terminal Schwann cells (De Winter et al., 2006) and muscle stem cells (Tatsumi et al., 2009). Furthermore, although motor nerves at the NMJ have been shown to express Npn1 protein (Venkova et al., 2014), many other cell types that reside within the skeletal muscle (such as the vasculature) express Npn1. Although we sought to better define Npn1 and Sema3A localization in the neuromuscular system, we could not achieve specific immunostaining with commercially available antibodies. Therefore, to directly examine if Sema3A signaling is required for reinnervation of the NMJ, we chose a strategy that allowed for the ubiquitous deletion of Npn1 (Fig. 4) or Sema3A (Fig. 5) from all adult tissues. Importantly, this allowed us to bypass complex expression patterns and circumvent the lack of tools required to demonstrate efficient, tissue-specific knockdown.

We first disrupted Sema3A signaling through the deletion of Npn1. As the high-affinity binding receptor for Sema3A, deletion of Npn1 renders cells insensitive to secreted Sema3A (He and Tessier-Lavigne, 1997; Kitsukawa et al., 1997; Kolodkin et al., 1997; Gu et al., 2003). *Npn1^fx/fx^* mice were crossed to the *Ubiquitin-C^CreERT2^*(*UBC^CreERT2^*) mouse line to generate *Cre*-negative littermates or *Cre*-positive conditional mutants (Npn1^UBC^). Tamoxifen (TMX) injections were then administered to initiate *Cre*-mediated recombination, and after a resting period of 12–15 d, a common peroneal nerve crush and contralateral sham injury were performed. EDL muscles were collected at various time points, and the extent of reinnervation was quantified (Fig. 4*A*). In some cases, Npn1^UBC^ mice were treated with corn oil (CO) and used as a vehicle control. Because *Cre*-negative and CO-treated Npn1^UBC^ mice behaved similarly by all parameters examined, both groups were combined into one Npn1^WT^ control littermate group. GP muscle and spinal cord tissue were collected from all mice examined in subsequent reinnervation analyses to validate that efficient and ubiquitous deletion of Npn1 was achieved. RT-qPCR demonstrated that Npn1^UBC^ mice had dramatically lower levels of *Npn1* expression than littermate controls (% knockdown: GP, 93.66% ± 1.20; SC, 91.44% ± 3.69; Fig. 4*B*). Furthermore, immunoprecipitation of Npn1 followed by immunoblotting showed that there was no detectable Npn1 protein in Npn1^UBC^ muscle (Fig. 4*C*) or spinal cord (Fig. 4*D*).

After global deletion of Npn1 in both spinal and muscle tissues was established, we quantified reinnervation at various time points after nerve crush. Similar to what we observed in wild-type mice (Fig. 1*E*), ∼200–300 NMJs were scored at the various time points in both Npn1^WT^ and Npn1^UBC^ mice (Fig. 4*E*). Additionally, analysis of sham-injured EDLs at the D21 time point found no significant differences in βIII-tubulin (Fig. 4*F*) or synapsin (Fig. 4*G*) scores between Npn1^WT^ and Npn1^UBC^ mice. Together, these results suggest that Npn1 is not integral for the postsynaptic apparatus after injury or for the normal maintenance of the NMJ.

To determine if Npn1 is involved in motor axon reinnervation, we examined βIII-tubulin (Fig. 4*H*, *I*) and synapsin (Fig. 4*J*, *K*) staining over the entire 50-d time course. Npn1^WT^ control littermates exhibited a pattern of reinnervation that closely resembled that observed in wild-type C57BL/6 mice (Fig. 1*F*, *G*). Furthermore, the deletion of Npn1 did not drastically alter the process of reinnervation in Npn1^UBC^ mice. However, at the D30 time point, we did observe a shift toward lower reinnervation scores, with Npn1^UBC^ mice exhibiting more partially innervated NMJs (Synapsin score 3: Npn1^WT^, 9.87% ± 2.50; Npn1^UBC^, 17.67% ± 2.38) and less fully innervated NMJs (Synapsin score 5: Npn1^WT^, 60.00% ± 8.08; Npn1^UBC^, 42.03% ± 2.65). To examine this more closely, the reinnervation data for scores 3–5 were replotted to directly compare Npn1^WT^ and Npn1^UBC^ genotypes (Fig. 4*L*, *M*). Examination of the data in this manner revealed that there is a significant reduction (**, *p* = 0.0037) of NMJs that received a fully reinnervated score of 5 based on synapsin staining at the D30 time point. Taken together, these results suggests that loss of Npn1 may slightly delay synaptic maturation of the motor nerve at the reinnervating NMJ. This delay, however, is only temporary, since both Npn1^UBC^ and Npn1^WT^ mice exhibit similar levels of presynaptic synapsin staining at D50 after injury.

In addition to examining reinnervation following Npn1 deletion, we also assessed the effect of Sema3A deletion on NMJ reinnervation. *Sema3A^fx/fx^* and *UBC^CreERT2^*mouse lines were crossed to generate *Cre*-negative littermates or *Cre*-positive conditional mutants (Sema3A^UBC^). TMX or CO injections were then administered to induce Cre-mediated recombination before performing a common peroneal nerve crush. RT-qPCR and immunoblotting were again used to examine Sema3A gene and protein expression. Sema3A^UBC^ mice treated with TMX exhibited significant knockdown of *Sema3A* transcript compared to Cre-negative control littermates (% knockdown: GP, 94.12% ± 0.69; SC, 89.29% ± 3.71). We also observed that CO-treated Sema3A^UBC^ mice showed a consistent, but partial reduction in *Sema3A* levels (% knockdown: GP, 57.91% ± 7.27; SC, 66.39% ± 7.74; Fig. 5*A*). Deletion of Sema3A protein was also demonstrated by immunoblotting. A protein band corresponding to the expected molecular weight of Sema3A (95–105 kDa) was detected in Sema3A^WT^ mice but absent in TMX-treated Sema3A^UBC^ GP muscle (Fig. 5*B*). Although the partial reduction of *Sema3A* mRNA in CO-treated Sema3A^UBC^ mice did not appear to lead to a reduction in Sema3A protein (Fig. 5*B*), we excluded that control group from subsequent reinnervation analysis. Importantly, Sema3A deletion before a denervating injury did not impact reinnervation of the NMJ. Loss of Sema3A did not alter the number of scored NMJs at the various time points examined (Fig. 5*C*). Furthermore, no differences in reinnervation were observed at any time point (Fig. 5*D*–*I*).

### Loss of Npn1 does not disrupt myelination or impair functional recovery after injury

**Figure 6.**
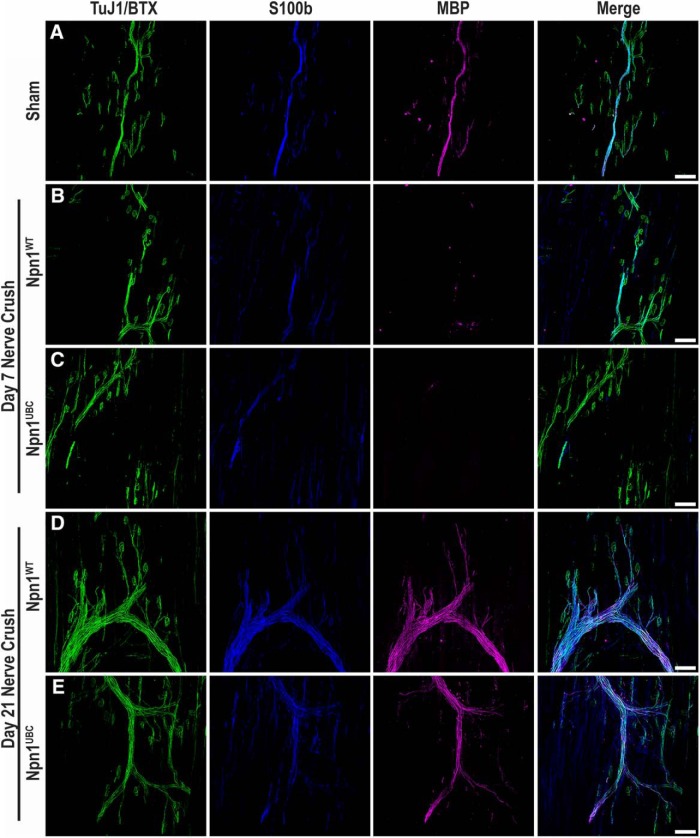
Npn1 deletion does not affect emyelination after a denervating injury. Myelination in sham-injured and denervated EDLs was examined. The nerve and endplates were visualized by staining for βIII-tubulin and BTX (green), while the myelin was visualized by staining for S100b (blue) and MBP (magenta). ***A***, Normal innervation and myelination is observed in sham-injured EDL sections. ***B***, ***C***, At D7 after nerve crush, there is a slight reduction in S100b and a near-total loss of MBP staining in both Npn1^WT^ (***B***) and Npn1^UBC^ (***C***) mice. ***D***, ***E***, Myelination was observed to have largely taken place by the D21 time point in both Npn1^WT^ (***D***) and Npn1^UBC^ (***E***) mice. Scale bars represent 100 µm.

Because it has been reported that terminal Schwann cells express Sema3A (De Winter et al., 2006), we explored whether the loss of Npn1 disrupted myelination of newly extended motor axons. Npn1^WT^ and Npn1^UBC^ EDL sections from the D7 and D21 time points were immunolabeled with βIII-tubulin and BTX (green) to visualize the motor nerve and the postsynaptic endplate region, while S100b (blue) and MBP (magenta) staining were used to visualize myelin proteins. Both innervation and myelination appeared normal in sham-injured sections (Fig. 6*A*), while varying degrees of NMJ reinnervation can be appreciated in both Npn1^WT^ (Fig. 6*B*) and Npn1^UBC^ mice (Fig. 6*C*) at the D7 time point after nerve crush. Furthermore, reinnervating motor axons of both genotypes exhibited a similar reduction in S100b immunofluorescence and the absence of MBP staining. By the D21 time point, Npn1^WT^ (Fig. 6*D*) and Npn1^UBC^ (Fig. 6*E*) motor axons displayed strong S100b and MBP immunofluorescence, indicating that the deletion of Npn1 does not affect the process of axonal myelination.

**Figure 7.**
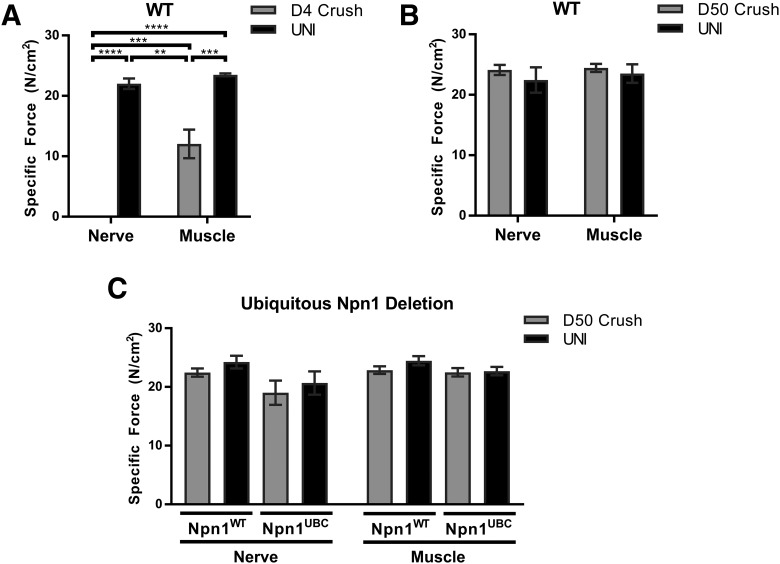
Functional recovery occurs normally after loss of Npn1. Specific force measurements after common peroneal nerve stimulation or direct EDL stimulation were examined in uninjured (UNI) mice and at D4 and D50 after nerve crush. ***A***, At the D4 time point, wild-type (WT) C57BL/6 mice (*n* = 3) demonstrate a loss of muscle force production after nerve stimulation and reduced force produced after muscle stimulation. ***B***, By the D50 time point, full functional recovery in WT mice (*n* = 5) is observed. ***C***, Npn1^UBC^ (*n* = 5) and Npn1^WT^ mice (*n* = 6) similarly demonstrate a full functional recovery after nerve crush at the D50 time point. Error bars represent the mean ± SE. ** *p* ≤ 0.01; ***, *p* ≤ 0.001; **** *p* ≤ 0.0001.

Finally, we performed muscle force measurements to examine functional recovery after the common peroneal nerve crush. First, C57BL/6 wild-type mice were used to demonstrate that we could effectively detect changes in muscle force production after injury. Briefly, at D4 after nerve crush, the common peroneal nerve and the EDL muscle were carefully exposed. The nerve and muscle were then independently stimulated to generate maximum isometric force (see Methods). As expected, the specific force produced by normally innervated uninjured contralateral EDL muscle was similar whether the contractions were elicited with nerve (22.01 ± 0.87 N/cm^2^) or muscle (23.49 ± 0.24 N/cm^2^) stimulation (Fig. 7*A*, black bars). At D4 after nerve crush (Fig. 7*A*, gray bars), there was no muscle force production generated after common peroneal nerve stimulation (0.02 ± 0.006 N/cm^2^), and there was a significant reduction in specific force compared with the contralateral control muscle during contractions produced by muscle stimulation (12.05 ± 2.36 N/cm^2^, *p* = 0.001). These results indicate that the nerve crush resulted in complete denervation of the EDL muscle, leading to alterations in the muscle that decreased force generating capacity by about half. Next, by assessing muscle force production at a later D50 time point, we demonstrated that C57BL/6 wild-type mice exhibited full re-innervation and functional recovery (Fig. 7*B*). Last, we examined functional recovery after ubiquitous Npn1 deletion. The specific force produced by the contralateral uninjured EDL was similar in both Npn1^UBC^ and Npn1^WT^ littermates after nerve or muscle stimulation (Fig. 7*C*, black bars), indicating that loss of Npn1 in adult mice does not directly alter neuromuscular activity. Furthermore, both Npn1^UBC^ and Npn1^WT^ mice demonstrated a full functional recovery at D50 after the common peroneal crush (Fig. 7*C*, gray bars).

## Discussion

It has long been appreciated that peripheral nerves exhibit a remarkable degree of plasticity and regenerative ability after injury. However, depending on the extent of the trauma after an acute injury and in cases of chronic denervation brought about by neurodegenerative diseases such as ALS, the capacity for peripheral nerve regeneration can be greatly limited. Consequently, failures in proper reinnervation of skeletal muscle lead to a reduction in neurotransmission, which in turn dampens the trophic support required for motor neuron and muscle fiber survival. Therefore, deciphering the molecular mechanism that underlies the regenerative response of motor axons has been a major focus within the field. Furthermore, it has been hypothesized that the exploitation of these pathways will yield improvements in motor axon regeneration that maximize muscle reinnervation to prevent functional deficits resulting from long-term denervation.

Although the regenerative response produced by denervation has already proven to be complex, many studies are beginning to tease apart the various signaling molecules that are likely playing key regulatory roles (Schmidt et al., 2009). Through its role as an axonal chemorepellent during development and its pattern of expression after injury at the NMJ, Sema3A signaling has been proposed to act as a negative regulator of motor axon reinnervation of the NMJ (De Winter et al., 2006). Additionally, work in other systems using Sema3A inhibitors has demonstrated that inhibition of Sema3A signaling improves regeneration after spinal cord transection (Kaneko et al., 2006) and enhances peripheral sensory/autonomic nerve regeneration in the cornea (Omoto et al., 2012). However, the functional consequence of altered Sema3A signaling on motor nerves that innervate the adult NMJ has not been previously examined.

Here, we fully characterized the regenerative response following a common peroneal nerve crush and demonstrated how it allows for the analysis of motor nerve reinnervation in a reproducible and highly quantifiable manner. Using this model, we directly examined how motor axon terminals respond to a denervating injury in the absence of Sema3A signaling by employing inducible knockout mice to delete Npn1, the high-affinity Sema3A-binding receptor, or Sema3A itself. Despite demonstrating that we could achieve a highly efficient and ubiquitous gene knockdown, perturbing Sema3A signaling did not alter the time course of muscle reinnervation or remyelination after injury.

While our study indicates that Sema3A signaling is not a necessary component of motor axon regeneration, we did observe some differences in *Sema3A* expression that conflict with what has previously been reported in other studies. Using a rat sciatic nerve crush model, De Winter et al. (2006) found that *Sema3A* is not normally expressed in uninjured skeletal muscle. However, after a denervating injury, they found a dramatic upregulation of *Sema3A* mRNA in fast-twitch gastrocnemius muscle fibers, while the same response was not observed in the predominantly slow-twitch soleus muscle. In contrast, we observed the opposite pattern of *Sema3A* expression in the fast-twitch EDL muscle after a common peroneal nerve crush. Namely, we could consistently detect *Sema3A* transcript in the uninjured adult EDL and we observed a significant decrease in *Sema3A* after nerve crush injury. Although it is not directly apparent what factor or factors underlie these discrepancies, it may be possible there are species-specific differences between the two models. Another possibility is that dorsoventral patterning may lead muscles of the posterior (gastrocnemius) and anterior (EDL) leg compartment to use different signaling pathways in different ways. Finally, it is also interesting to note that data from both studies similarly suggests that Sema3A signaling may be playing a larger role in fast-twitch muscle fibers. Although we did not examine how *Sema3A* levels are altered in the soleus muscle after injury, in uninjured muscle we found ∼3–4-fold lower levels of *Sema3A* mRNA compared to that observed in the fast-twitch GP and EDL muscles. What accounts for differences in *Sema3A* expression in different muscle groups and the significance of those differences, if any, remains an open question.

Recently, it has been proposed that Npn1-Sema3A signaling may play a role at the postnatal NMJ (Helmbrecht et al., 2015; Saller et al., 2016). However, for those studies, the authors used the *Olig2* promoter to conditionally delete Npn1 from motor neurons. Because the *Olig2* promoter turns on very early in development (Masahira et al., 2006), it is not possible to resolve whether the observed phenotype is due to an earlier axon pathfinding defect or the loss of active Npn1-Sema3A signaling at the NMJ. Therefore, to our knowledge, this is the first published study to directly examine how the adult NMJ responds to the absence of Sema3A signaling. We addressed this question by examining innervation in sham-injured EDLs at the D21 time point and overall found no effect on the NMJ histology after Npn1 deletion. Both the total number of scored NMJs and their innervation status (as scored by nerve βIII-tubulin and the presynaptic marker synapsin) were similar among Npn1^UBC^ and Npn1^WT^ mice. It is important to note that while this analysis was performed at the D21 time point, we can estimate that Npn1 was ubiquitously deleted for ∼1 mo (see Fig. 4*A*). Furthermore, from previous studies, it is known that acetylcholine receptors in uninjured skeletal muscle have a half turnover rate of ∼10 d (Loring and Salpeter, 1980). Therefore, enough time should have elapsed to determine if Npn1 plays a major role in the maintenance of the NMJ.

An interesting observation we made following Npn1 deletion was that at the D30 time point, there was a statistically significant decrease in the number of NMJs that received the highest innervation score based on synapsin staining. However, the same effect was not observed after Sema3A deletion. This, combined with our finding that *Npn1* levels decrease after nerve crush and do not return to uninjured levels until the D30 time point, suggest that signaling through the Npn1 may play a minor role in the maturation of the presynaptic motor nerve after injury. It is also noteworthy that these observations fit with recently published studies of Npn1-Sema3A signaling at the NMJ. More specifically, a pharmacological inhibition of the Npn1 receptor was shown to delay denervation and prolong lifespan in an ALS mouse model (Venkova et al., 2014), while mice engineered to express a mutated form of Sema3A protein did not display any defects in response to denervation or the neurodegenerative disease process (Moloney et al., 2017).

Taken together, these data suggest that Sema3A signaling is dispensable in the context of adult motor nerve regeneration. However, signaling through the Npn1 receptor may play a transient role in mediating NMJ synaptic maturation. Interestingly, Npn1 contains multiple extracellular domains that have been suggested to mediate a diverse array of protein interactions (Fujisawa et al., 1997; Nakamura and Goshima, 2002). For example, the vascular endothelial growth factor (VEGF) pathway has been well established to exert its signaling actions through the Npn1 receptor (Soker et al., 1998). Additionally, VEGF signaling has also been found to be protective against neurodegeneration in ALS mouse models (Storkebaum et al., 2005; Zheng et al., 2007), suggesting that VEGF-Npn1 signaling is an avenue that warrants further exploration. Finally, one intriguing possibility is that there is a compensatory pathway activated in the absence of Sema3A-Npn1 signaling. While we cannot specifically rule out this possibility, our work suggests that direct targeting of these pathways will not be a substantial avenue for future therapeutic research on nerve regeneration after injury. However, by continuing to better understand the complex processes that regulate muscle reinnervation, we move closer to uncovering new strategies to promote motor axon regeneration and prevent functional deficits associated with both injury and disease.

## Synthesis

Reviewing Editor: Robert Kalb, Northwestern University Decisions are customarily a result of the Reviewing Editor and the peer reviewers coming together and discussing their recommendations until a consensus is reached. When revisions are invited, a fact-based synthesis statement explaining their decision and outlining what is needed to prepare a revision will be listed below. The following reviewer(s) agreed to reveal their identity: Fumio Nakamura

Both reviewers favor publication after correction of a few minor items:

Rev 1

One minor comment about citation:

Tan et al, 2011 and Fukuda et al., 2013 in PCR sequence table are missing in Reference list. Please include these articles in the list.

Rev 2

Sema3A signalliing is believed to be transmitted by flt/flk receptors as well as by NPn1. That literature would be worth a few lines in the manuscript.

Figure 5 is poorly labelled, and this reviewer found it to be very difficult to interpret for that reason.

The authors did finally manage to get a Sema3A antibody to work. Would that antibody also permit proof of the Sema3A KO in the peripheral nerve, and to determine whether, in fact, Sema3A does go up in the nerve post-crush?

Some Sema3A inhibitors have been reported, and there is one publication (Omoto et al, PLoS One 7:e47716, 2012, that claims that that inhibitor accelerates peripheral nerve regeneration in cornea. That paper should be cited and discussed.

## References

[B1] Agbulut O, Noirez P, Beaumont F, Butler-Browne G (2003) Myosin heavy chain isoforms in postnatal muscle development of mice. Biol Cell 95:399–406. 14519557 10.1016/s0248-4900(03)00087-x

[B2] Ara J, Bannerman P, Hahn A, Ramirez S, Pleasure D (2004) Modulation of sciatic nerve expression of class 3 semaphorins by nerve injury. Neurochem Res 29:1153–1159. 15176472 10.1023/b:nere.0000023602.72354.82

[B3] Bagri A, Cheng HJ, Yaron A, Pleasure SJ, Tessier-Lavigne M (2003) Stereotyped pruning of long hippocampal axon branches triggered by retraction inducers of the semaphorin family. Cell 113:285–299. 12732138 10.1016/s0092-8674(03)00267-8

[B4] Bauder AR, Ferguson TA (2012) Reproducible mouse sciatic nerve crush and subsequent assessment of regeneration by whole mount muscle analysis. J Vis Exp (60):3606. 10.3791/3606 PMC3376939 22395197

[B5] Behar O, Golden JA, Mashimo H, Schoen FJ, Fishman MC (1996) Semaphorin III is needed for normal patterning and growth of nerves, bones and heart. Nature 383:525–528. 10.1038/383525a0 8849723

[B6] Bowen DC, Park JS, Bodine S, Stark JL, Valenzuela DM, Stitt TN, Yancopoulos GD, Lindsay RM, Glass DJ, DiStefano PS (1998) Localization and regulation of MuSK at the neuromuscular junction. Dev Biol 199:309–319. 10.1006/dbio.1998.8936 9698449

[B7] Brooks SV, Faulkner JA (1988) Contractile properties of skeletal muscles from young, adult and aged mice. J Physiol 404:71–82. 3253447 10.1113/jphysiol.1988.sp017279 PMC1190815

[B8] Cheadle L, Biederer T (2014) Activity-dependent regulation of dendritic complexity by semaphorin 3A through Farp1. J Neurosci 34:7999–8009. 10.1523/JNEUROSCI.3950-13.2014 24899721 PMC4044256

[B9] Chen ZL, Yu WM, Strickland S (2007) Peripheral regeneration. Annu Rev Neurosci 30:209–233. 10.1146/annurev.neuro.30.051606.094337 17341159

[B10] Dalkin W, Taetzsch T, Valdez G (2016) The fibular nerve injury method: a reliable assay to identify and test factors that repair neuromuscular junctions. J Vis Exp (114). 10.3791/54186 PMC5091792 27585036

[B11] De Winter F, Vo T, Stam FJ, Wisman LA, Bär PR, Niclou SP, van Muiswinkel FL, Verhaagen J (2006) The expression of the chemorepellent Semaphorin 3A is selectively induced in terminal Schwann cells of a subset of neuromuscular synapses that display limited anatomical plasticity and enhanced vulnerability in motor neuron disease. Mol Cell Neurosci 32:102–117. 10.1016/j.mcn.2006.03.002 16677822

[B12] Dent EW, Barnes AM, Tang F, Kalil K (2004) Netrin-1 and semaphorin 3A promote or inhibit cortical axon branching, respectively, by reorganization of the cytoskeleton. J Neurosci 24:3002–3012. 10.1523/JNEUROSCI.4963-03.2004 15044539 PMC6729836

[B13] Duchen LW (1970) Changes in motor innervation and cholinesterase localization induced by botulinum toxin in skeletal muscle of the mouse: differences between fast and slow muscles. J Neurol Neurosurg Psychiatry 33:40–54. 4907278 10.1136/jnnp.33.1.40 PMC493406

[B14] Fawcett JW, Keynes RJ (1990) Peripheral nerve regeneration. Annu Rev Neurosci 13:43–60. 10.1146/annurev.ne.13.030190.000355 2183684

[B15] Frey D, Schneider C, Xu L, Borg J, Spooren W, Caroni P (2000) Early and selective loss of neuromuscular synapse subtypes with low sprouting competence in motoneuron diseases. J Neurosci 20:2534–2542. 10729333 10.1523/JNEUROSCI.20-07-02534.2000 PMC6772256

[B16] Fujisawa H, Kitsukawa T, Kawakami A, Takagi S, Shimizu M, Hirata T (1997) Roles of a neuronal cell-surface molecule, neuropilin, in nerve fiber fasciculation and guidance. Cell Tissue Res 290:465–470. 9321711 10.1007/s004410050954

[B17] Fukuda T, et al. (2013) Sema3A regulates bone-mass accrual through sensory innervations. Nature 497:490–493. 10.1038/nature12115 23644455

[B18] Gu C, Rodriguez ER, Reimert DV, Shu T, Fritzsch B, Richards LJ, Kolodkin AL, Ginty DD (2003) Neuropilin-1 conveys semaphorin and VEGF signaling during neural and cardiovascular development. Dev Cell 5:45–57. 12852851 10.1016/s1534-5807(03)00169-2 PMC3918747

[B19] Gumerson JD, Davis CS, Kabaeva ZT, Hayes JM, Brooks SV, Michele DE (2013) Muscle-specific expression of LARGE restores neuromuscular transmission deficits in dystrophic LARGE(myd) mice. Hum Mol Genet 22:757–768. 10.1093/hmg/dds483 23222475 PMC3554202

[B20] Gupta SK, Poduslo JF, Mezei C (1988) Temporal changes in PO and MBP gene expression after crush-injury of the adult peripheral nerve. Brain Res 464:133–141. 2464407 10.1016/0169-328x(88)90005-8

[B21] Haupt C, Kloos K, Faus-Kessler T, Huber AB (2010) Semaphorin 3A-neuropilin-1 signaling regulates peripheral axon fasciculation and pathfinding but not developmental cell death patterns. Eur J Neurosci 31:1164–1172. 10.1111/j.1460-9568.2010.07154.x 20345923

[B22] He Z, Tessier-Lavigne M (1997) Neuropilin is a receptor for the axonal chemorepellent Semaphorin III. Cell 90:739–751. 9288753 10.1016/s0092-8674(00)80534-6

[B23] Helmbrecht MS, Soellner H, Truckenbrodt AM, Sundermeier J, Cohrs C, Hans W, de Angelis MH, Feuchtinger A, Aichler M, Fouad K, Huber AB (2015) Loss of Npn1 from motor neurons causes postnatal deficits independent from Sema3A signaling. Dev Biol 399:2–14. 10.1016/j.ydbio.2014.11.024 25512301

[B24] Huber AB, Kania A, Tran TS, Gu C, De Marco Garcia N, Lieberam I, Johnson D, Jessell TM, Ginty DD, Kolodkin AL (2005) Distinct roles for secreted semaphorin signaling in spinal motor axon guidance. Neuron 48:949–964. 10.1016/j.neuron.2005.12.003 16364899

[B25] Janssen BJ, Malinauskas T, Weir GA, Cader MZ, Siebold C, Jones EY (2012) Neuropilins lock secreted semaphorins onto plexins in a ternary signaling complex. Nat Struct Mol Biol 19:1293–1299. 10.1038/nsmb.2416 23104057 PMC3590443

[B26] Kaneko S, Iwanami A, Nakamura M, Kishino A, Kikuchi K, Shibata S, Okano HJ, Ikegami T, Moriya A, Konishi O, Nakayama C, Kumagai K, Kimura T, Sato Y, Goshima Y, Taniguchi M, Ito M, He Z, Toyama Y, Okano H (2006) A selective Sema3A inhibitor enhances regenerative responses and functional recovery of the injured spinal cord. Nat Med 12:1380–1389. 10.1038/nm1505 17099709

[B27] Kanning KC, Kaplan A, Henderson CE (2010) Motor neuron diversity in development and disease. Annu Rev Neurosci 33:409–440. 10.1146/annurev.neuro.051508.135722 20367447

[B28] Kitsukawa T, Shimizu M, Sanbo M, Hirata T, Taniguchi M, Bekku Y, Yagi T, Fujisawa H (1997) Neuropilin-semaphorin III/D-mediated chemorepulsive signals play a crucial role in peripheral nerve projection in mice. Neuron 19:995–1005. 10.1016/S0896-6273(00)80392-X 9390514

[B29] Kolodkin AL, Levengood DV, Rowe EG, Tai YT, Giger RJ, Ginty DD (1997) Neuropilin is a semaphorin III receptor. Cell 90:753–762. 9288754 10.1016/s0092-8674(00)80535-8

[B30] Li FQ, Fowler KA, Neil JE, Colton CA, Vitek MP (2010) An apolipoprotein E-mimetic stimulates axonal regeneration and remyelination after peripheral nerve injury. J Pharmacol Exp Ther 334:106–115. 10.1124/jpet.110.167882 20406857 PMC2912037

[B31] Loring RH, Salpeter MM (1980) Denervation increases turnover rate of junctional acetylcholine receptors. Proc Natl Acad Sci U S A 77:2293–2297. 6929550 10.1073/pnas.77.4.2293 PMC348700

[B32] Lowrie MB, Krishnan S, Vrbová G (1982) Recovery of slow and fast muscles following nerve injury during early post-natal development in the rat. J Physiol 331:51–66. 7153915 10.1113/jphysiol.1982.sp014364 PMC1197741

[B33] Luo Y, Raible D, Raper JA (1993) Collapsin: a protein in brain that induces the collapse and paralysis of neuronal growth cones. Cell 75:217–227. 8402908 10.1016/0092-8674(93)80064-l

[B34] Magill CK, Tong A, Kawamura D, Hayashi A, Hunter DA, Parsadanian A, Mackinnon SE, Myckatyn TM (2007) Reinnervation of the tibialis anterior following sciatic nerve crush injury: a confocal microscopic study in transgenic mice. Exp Neurol 207:64–74. 10.1016/j.expneurol.2007.05.028 17628540 PMC2000860

[B35] Masahira N, Takebayashi H, Ono K, Watanabe K, Ding L, Furusho M, Ogawa Y, Nabeshima Y, Alvarez-Buylla A, Shimizu K, Ikenaka K (2006) Olig2-positive progenitors in the embryonic spinal cord give rise not only to motoneurons and oligodendrocytes, but also to a subset of astrocytes and ependymal cells. Dev Biol 293:358–369. 10.1016/j.ydbio.2006.02.029 16581057

[B36] Messersmith EK, Leonardo ED, Shatz CJ, Tessier-Lavigne M, Goodman CS, Kolodkin AL (1995) Semaphorin III can function as a selective chemorepellent to pattern sensory projections in the spinal cord. Neuron 14:949–959. 7748562 10.1016/0896-6273(95)90333-x

[B37] Mitchell LS, Griffiths IR, Morrison S, Barrie JA, Kirkham D, McPhilemy K (1990) Expression of myelin protein gene transcripts by Schwann cells of regenerating nerve. J Neurosci Res 27:125–135. 10.1002/jnr.490270202 1701490

[B38] Moloney EB, de Winter F, Verhaagen J (2014) ALS as a distal axonopathy: molecular mechanisms affecting neuromuscular junction stability in the presymptomatic stages of the disease. Front Neurosci 8:252. 10.3389/fnins.2014.00252 25177267 PMC4132373

[B39] Moloney EB, Hobo B, De Winter F, Verhaagen J (2017) Expression of a mutant SEMA3A protein with diminished signalling capacity does not alter ALS-related motor decline, or confer changes in NMJ plasticity after BotoxA-induced paralysis of male gastrocnemic muscle. PLoS One 12:e0170314. 10.1371/journal.pone.0170314 28103314 PMC5245795

[B40] Morita A, Yamashita N, Sasaki Y, Uchida Y, Nakajima O, Nakamura F, Yagi T, Taniguchi M, Usui H, Katoh-Semba R, Takei K, Goshima Y (2006) Regulation of dendritic branching and spine maturation by semaphorin3A-Fyn signaling. J Neurosci 26:2971–2980. 10.1523/JNEUROSCI.5453-05.2006 16540575 PMC6673984

[B41] Nakamura F, Goshima Y (2002) Structural and functional relation of neuropilins. Adv Exp Med Biol 515:55–69. 12613543 10.1007/978-1-4615-0119-0_5

[B42] Omoto M, Yoshida S, Miyashita H, Kawakita T, Yoshida K, Kishino A, Kimura T, Shibata S, Tsubota K, Okano H, Shimmura S (2012) The semaphorin 3A inhibitor SM-345431 accelerates peripheral nerve regeneration and sensitivity in a murine corneal transplantation model. PLoS One 7:e47716. 10.1371/journal.pone.0047716 23152758 PMC3494696

[B43] Rohm B, Ottemeyer A, Lohrum M, Püschel AW (2000) Plexin/neuropilin complexes mediate repulsion by the axonal guidance signal semaphorin 3A. Mech Dev 93:95–104. 10781943 10.1016/s0925-4773(00)00269-0

[B44] Saller MM, Huettl RE, Hanuschick P, Amend AL, Alberton P, Aszodi A, Huber AB (2016) The role of Sema3-Npn-1 signaling during diaphragm innervation and muscle development. J Cell Sci 129:3295–3308. 10.1242/jcs.186015 27466379 PMC5047703

[B45] Scarlato M, Ara J, Bannerman P, Scherer S, Pleasure D (2003) Induction of neuropilins-1 and -2 and their ligands, Sema3A, Sema3F, and VEGF, during Wallerian degeneration in the peripheral nervous system. Exp Neurol 183:489–498. 14552889 10.1016/s0014-4886(03)00046-3

[B46] Schmidt ER, Pasterkamp RJ, van den Berg LH (2009) Axon guidance proteins: novel therapeutic targets for ALS?. Prog Neurobiol 88:286–301. 10.1016/j.pneurobio.2009.05.004 19523502

[B47] Shelly M, Cancedda L, Lim BK, Popescu AT, Cheng PL, Gao H, Poo MM (2011) Semaphorin3A regulates neuronal polarization by suppressing axon formation and promoting dendrite growth. Neuron 71:433–446. 10.1016/j.neuron.2011.06.041 21835341 PMC3164872

[B48] Soker S, Takashima S, Miao HQ, Neufeld G, Klagsbrun M (1998) Neuropilin-1 is expressed by endothelial and tumor cells as an isoform-specific receptor for vascular endothelial growth factor. Cell 92:735–745. 9529250 10.1016/s0092-8674(00)81402-6

[B49] Storkebaum E, et al (2005) Treatment of motoneuron degeneration by intracerebroventricular delivery of VEGF in a rat model of ALS. Nat Neurosci 8:85–92. 10.1038/nn1360 15568021

[B50] Takahashi T, Fournier A, Nakamura F, Wang LH, Murakami Y, Kalb RG, Fujisawa H, Strittmatter SM (1999) Plexin-neuropilin-1 complexes form functional semaphorin-3A receptors. Cell 99:59–69. 10520994 10.1016/s0092-8674(00)80062-8

[B51] Tan KY, Eminli S, Hettmer S, Hochedlinger K, Wagers AJ (2011) Efficient generation of iPS cells from skeletal muscle stem cells. PLoS One 6:e26406. 10.1371/journal.pone.0026406 22028872 PMC3196574

[B52] Taniguchi M, Yuasa S, Fujisawa H, Naruse I, Saga S, Mishina M, Yagi T (1997) Disruption of semaphorin III/D gene causes severe abnormality in peripheral nerve projection. Neuron 19:519–530. 10.1016/S0896-6273(00)80368-2 9331345

[B53] Tatsumi R, Sankoda Y, Anderson JE, Sato Y, Mizunoya W, Shimizu N, Suzuki T, Yamada M, Rhoads RP, Jr., Ikeuchi Y, Allen RE (2009) Possible implication of satellite cells in regenerative motoneuritogenesis: HGF upregulates neural chemorepellent Sema3A during myogenic differentiation. Am J Physiol Cell Physiol 297:C238–C252. 10.1152/ajpcell.00161.2009 19515904

[B54] Valenzuela DM, Stitt TN, DiStefano PS, Rojas E, Mattsson K, Compton DL, Nuñez L, Park JS, Stark JL, Gies DR, et al. (1995) Receptor tyrosine kinase specific for the skeletal muscle lineage: expression in embryonic muscle, at the neuromuscular junction, and after injury. Neuron 15:573–584. 7546737 10.1016/0896-6273(95)90146-9

[B55] Venkova K, Christov A, Kamaluddin Z, Kobalka P, Siddiqui S, Hensley K (2014) Semaphorin 3A signaling through neuropilin-1 is an early trigger for distal axonopathy in the SOD1G93A mouse model of amyotrophic lateral sclerosis. J Neuropathol Exp Neurol 73:702–713. 10.1097/NEN.0000000000000086 24918638 PMC4072440

[B56] Xu QG, Midha R, Martinez JA, Guo GF, Zochodne DW (2008) Facilitated sprouting in a peripheral nerve injury. Neuroscience 152:877–887. 10.1016/j.neuroscience.2008.01.060 18358630

[B57] Yaron A, Zheng B (2007) Navigating their way to the clinic: emerging roles for axon guidance molecules in neurological disorders and injury. Dev Neurobiol 67:1216–1231. 10.1002/dneu.20512 17514715

[B58] Yaron A, Huang PH, Cheng HJ, Tessier-Lavigne M (2005) Differential requirement for Plexin-A3 and -A4 in mediating responses of sensory and sympathetic neurons to distinct class 3 Semaphorins. Neuron 45:513–523. 10.1016/j.neuron.2005.01.013 15721238

[B59] Yazdani U, Terman JR (2006) The semaphorins. Genome Biol 7:211 10.1186/gb-2006-7-3-211 16584533 PMC1557745

[B60] Zheng C, Sköld MK, Li J, Nennesmo I, Fadeel B, Henter JI (2007) VEGF reduces astrogliosis and preserves neuromuscular junctions in ALS transgenic mice. Biochem Biophys Res Commun 363:989–993. 10.1016/j.bbrc.2007.09.088 17923114

